# An outbreak of acute norovirus gastroenteritis in a boarding school in Shanghai: a retrospective cohort study

**DOI:** 10.1186/1471-2458-14-1092

**Published:** 2014-10-22

**Authors:** Caoyi Xue, Yifei Fu, Weiping Zhu, Yi Fei, Linying Zhu, Hong Zhang, Lifeng Pan, Hongmei Xu, Yong Wang, Wenqin Wang, Qiao Sun

**Affiliations:** Research Base of Key Laboratory of Surveillance and Early warning on Infectious Disease in China CDC, Shanghai Pudong District Center for Disease Control and Prevention, 3039 Zhangyang Road, Shanghai, 200136 China; Department of Infectious Disease, Pudong District Center for Disease Control and Prevention, Shanghai, China

**Keywords:** Norovirus, Acute gastroenteritis, Outbreak, Asymptomatic food handler

## Abstract

**Background:**

More than 200 students and teachers at a boarding school in Shanghai developed acute gastroenteritis in December, 2012. The transmission mode remained largely unknown. An immediate epidemiological investigation was conducted to identify it.

**Methods:**

Using a retrospective cohort design, we investigated demographic characteristics, school environment, and previous contacts with people who had diarrhea and/or vomiting, drinking water conditions, recalls of food consumption in the school cafeteria, hand-washing habits and eating habits. Rectal swabs of the new cases and food handlers as well as water and food samples were collected to test potential bacteria and viruses. *Norovirus* was detected by real-time reverse transcription-polymerase chain reaction (RT-PCR).

**Results:**

A total of 278 cases developed gastrointestinal symptoms in this outbreak, and the overall attack rate was 13.9%. The main symptoms included vomiting (50.0%), abdominal cramps (40.3%), nausea (27.0%), diarrhea (6.8%) and fever (6.8%). Twenty rectal swab samples were detected as *Norovirus*–positive, including 11 from student cases and 9 from asymptomatic food handlers (non-cases). Among environmental surface samples from the kitchen, 8 samples were also detected as *Norovirus*-positive. The genotypes of viral strains were the same (GII) in patients, asymptomatic food handlers and environmental surfaces. Other samples, including rectal swabs, water samples and food samples were negative for any bacteria and other tested viruses. Asymptomatic food handlers may have contaminated the cooked food during the food preparation.

**Conclusion:**

The study detected that the outbreak was caused by *Norovirus* and should be controlled by thorough disinfection and excluding asymptomatic food handlers from food preparation. Early identification of the predominant mode of transmission in this outbreak was necessary to prevent new cases. Furthermore, good hygiene practices such as regular hand washing and efficient daily disinfection should be promoted to prevent such infection and outbreaks.

## Background

*Norovirus* is known as the most common cause of acute gastroenteritis among adults and children and is having a significant influence on public health [1]. A systematic review and meta-analysis recently published in the *Lancet* found that the estimated pooled prevalence of *Norovirus* in patients with acute gastroenteritis was 18% (95% CI: 17–20). The prevalence of *Norovirus* in cases of acute gastroenteritis tended to be higher in community and outpatient settings compared with inpatient settings. The prevalence was also higher in developing and developed countries with low mortalities compared with developing countries with high mortalities [2]. Infectious non-bacterial gastroenteritis caused by *Norovirus* leads to a worldwide epidemic with infection rates peaking during the winter months [3]. The symptoms usually begin after a12-48 hour incubation period and are characterized by an acute onset, non-bloody diarrhea, vomiting, nausea and abdominal cramps, all of which might be severe and typically disappear after 1–3 days without treatment. Children and the elderly may take up to 4–6 days to recover [4]. *Norovirus* can be spread by 3 main routes, including contaminated food or water, person-to-person contacts and aerosol [5]. *Norovirus* is highly infectious and can be easily transmitted in many ways, such as contact with infectious individuals, contact with contaminated agents in the environment and consuming contaminated food or water [6–8].

*Noroviruses* can be divided into at least five genogroups, designated GI–GV, which are based on the amino acid found in the major structural protein (VP1). The strains that infect humans (referred to collectively as “human *Noroviruses*”) are found in the GI, GII, and GIV genogroups, whereas the strains infecting cows and mice are found in GIII and GV, respectively [9]. *Noroviruses* can be classified further into genotypes with at least eight genotypes belonging to GI and 21 genotypes belonging to GII [5]. Since 2001, GII.4 viruses have been associated with the majority of viral gastroenteritis outbreaks worldwide [3]. Recent studies have found that these viruses have evolved through serial changes in the VP1 sequence over time, which allowed evasion of immunity in the human population [3, 10]. Furthermore, patient age and whether the years that a novel strain emerged were included were not associated with population prevalence of *Norovirus* [2] *.*

Based on our diarrhea syndromic surveillance system covering 12 sentinel hospitals in the Pudong district of Shanghai [11], we found that *Norovirus* has accounted for nearly 60% of non-bacterial diarrhea cases with a peak incidence in the winter season.

On November 21^st^ 2012, the Pudong District Center for Disease Control and Prevention (PDCDC) was notified that more than 100 students at a boarding school had developed symptoms of diarrhea and vomiting within 3 days. To control this outbreak, the PDCDC immediately formed a team to conduct an epidemiological investigation at the school.

## Methods

### Study setting

The boarding school was a private school from the 1^st^ to 12^th^ grades with a total of 58 classes, 1693 students and 302 staff, including both teachers and cafeteria food handlers. Of the student body, 1373 students lived in the school dormitory. The school only had 1 cafeteria in which all the boarding students, some on-duty teachers and cafeteria food handlers had 3 meals daily. Other teachers and day students only had lunch in the cafeteria. All of the meals were cooked in the kitchen of the school cafeteria. The cafeteria provided freshly prepared and completely cooked meals every day for the students and teachers. The Shanghai Food and Drug Administration (Shanghai FDA) requires that school cafeterias keep a food sample for 48 hours in one fixed refrigerator for each meal and that raw foods be stored in another refrigerator in a separate room. Students in grades 7–12 and all staff had meals in the cafeteria, while students in grades 1–6 had meals delivered by food handlers in their classroom. One-fourth of the 50 food handlers were in charge of distributing the cooked meals. The cafeteria food handlers implemented regular daily shifts and rotated shifts for weekends. All foods were served as usual from November 18^th^ to November 21^st^.

The school provided bottled drinking water manufactured by a drinking water factory. Before the outbreak, the most recent test for drinking water quality was conducted on Oct 22^nd^, 2012 and was accredited as meeting the Drinking Water Standard (GB5749-2006).

### Epidemiological investigation

We designed a retrospective cohort study to conduct the investigation and to analyze the potential transmission mode. In this outbreak, cases were classified as probable cases and laboratory-confirmed cases. Probable cases were students or staff in the boarding school with at least 1 of the following 4 symptoms: 1) diarrhea (watery or mushy stool) 3 or more times within 24 hours, 2) vomiting, 3) nausea, and 4) abdominal cramps from November 18^th^ to December 7^th^ 2012. Laboratory-confirmed cases were the probable cases whose rectal swabs tested positive for *Norovirus* using a real-time RT-PCR. We searched for cases among all students, teachers and other staff members based on an epidemiological questionnaire of gastrointestinal (GI) symptoms. The questionnaire was used to collect information on demographic characteristics, GI symptoms, meals in the school cafeteria, sources of drinking water, history of contact with persons who had diarrhea and/or vomiting, and personal hygiene habits. We also collected information on the school cafeteria’s hygiene condition, health certificates of cafeteria food handlers and a test report for drinking water quality. The study was conducted according to the principles and guidelines of the Declaration of Helsinki and was approved by the Research Ethics Committee at the PDCDC.

### Specimen collection

Rectal swabs were selected to be the first collection method for clinical specimens to promptly identify the outbreak, because some cases who had no symptoms of diarrhea had difficulty providing stool or vomitus specimens. Table 1 shows the detailed specimen collections from November 22^nd^ - December 6^th^ during this outbreak. Due to the potential key role of school cafeteria food handlers in transmitting the intestinal virus, we also collected 10 rectal swabs of on-duty food handlers who were mainly engaged in the cooking and distribution during the first field investigation on November 22^nd^, even though none of them had reported any similar symptoms. Rectal swabs from the remainder of the food handlers were also collected on November 23^rd^. Food samples from the last 2 days (November 19^th^-20^th^) were provided by the school cafeteria, and drinking-water samples were also collected on November 22^nd^. For highly transmissible environmental contagious agents, 153 specimens of common surfaces that spread pathogens through a rectal-oral route, such as doorknobs, toilets taps, kitchen rags, kitchen cabinet, cafeteria tables, food carts, cutlery, classroom tables and taps of drinking water machine taps were collected on November 22^nd^. All samples were transported to the PDCDC laboratory via bio-safety cycle boxes and transfer vehicles, which were disinfected after use to detect intestinal bacteria including *E. coli, Salmonella, Shigella, Yersinia enterocolitica, Vibrio cholerae, Vibrio parahaemolyticus, Aeromonas hydrophila* and *Plesiomonas Shigelloides* and intestinal viruses including *Rotavirus, Enteric adenovirus, Norovirus, Saporovirus* and *Aastrovirus.* The bacterial and viral pathogens were tested according to the technical procedures of diarrheal pathogenic spectrum surveillance formulated by the China CDC to process bacterial isolation, culture and purification. The identification of species was conducted using system biochemical slats [12].

**Table 1:** **Total specimens for laboratory test during the outbreak investigation**

Sampling subjects	No. of specimens	Sampling date	No. of positive specimens
Cases	12	November 22^nd^	2
	5	November 24^th^	1
	4	November 25^th^	1
	1	November 26^th^	0
	8	November 28^th^	2
	3	November 29^th^	1
	3	November 30^th^	2
	2	December 3^rd^	2
Asymptomatic food handlers*	10	November 22^nd^	4
	40	November 23^rd^	5
Environment surfaces	153	November 22^nd^	8
Drinking water	2	November 22^nd^	0
Food samples	10	November 22^nd^	0

*The repeated tests of food handlers were not included.

### Sample processing and RNA extraction

Approximately 0.2 g of stool samples was added to a 5 mL eppendorf tube, which contained 2 mL pH 7.2 phosphate buffered saline (PBS). After being vortexed for 30 sec, samples were clarified by centrifugation at 8,000 × g for 5 min at room temperature. Nucleic acid was extracted from the suspension. Nucleic acid was extracted from a 250 μl suspension using the NucliSens easyMAG platform with the NucliSens magnetic extraction reagents (bioMe´rieux, The Netherlands) according to the manufacturer’s instruction. The elution volume of nucleic acid was 50 μl.

### Real-time fluorescence RT-PCR

*Rotavirus* (Lot NO, DD-0044-02) and *Norovirus* (Lot NO, DD-0045-02) detection kits were produced by Shanghai ZJ Bio-Tech CO., LTD (Shanghai,China). According to the manufacturer’s instruction, 5 μl nucleic acid was added to the Master Mix, which consisted of 18 μl Super Mix, 1 μl Enzyme Mix and 1 μl Internal Control. Real-time PCR was performed using an ABI 7500 real-time PCR system at 45°C for 10 min for 1 cycle, 95°C for 15 min for 1 cycle, 95°C for 15 sec, and 60°C for 60 sec for 40 cycles. Fluorescence was measured at 60°C, and a FAM channel was chosen. If the Ct value in channel showed ≤38, the result was positive. If the Ct value in channel was between 38–40, then it was repeated. If the result was still 38–40, the result was considered to be negative.

### Statistical analysis

The distributions of the major symptoms in the outbreaks were summarized using frequencies and proportions. Attack rates were calculated to assess the association between the associated factors and disease. *Chi-square* tests of the attack rate in the different groups were performed. All statistical tests were 2-sided, and P values <0.05 were considered to be statistically significant.

## Results

A total of 278 cases developed gastrointestinal symptoms during this outbreak, and the attack rate was 13.9%. Vomiting and abdominal cramps were the main symptoms. The attack rate in students in grades 1–6 (elementary school) was much higher than in those in grades 7-12 (middle and high school). The students who had 3 meals a day in the school and students in grades 1–6 had higher probabilities of infection. A total of 20 rectal swab samples were detected as *Norovirus*-positive, including 11 from student cases and 9 from asymptomatic food handlers (non-cases). Eight environmental surface samples, all in the kitchen, were also detected as *Norovirus*-positive. The genotypes of viral strains were the same (GII) in patients, asymptomatic food handlers and environmental surfaces. The PDCDC used immediate control measures including surveillance, disinfection and removing asymptomatic food handlers to stop this outbreak.

### Descriptive statistics

**Figure 1:**
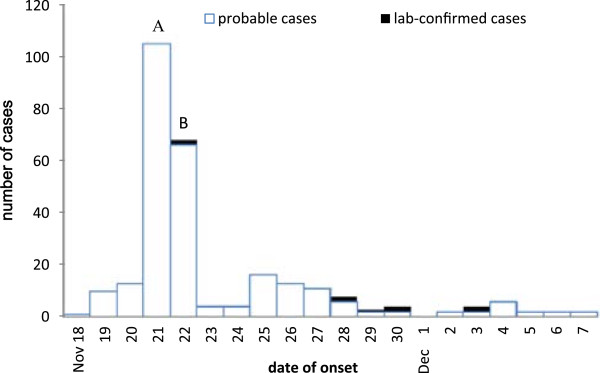
**Time distribution of the outbreak: epidemic curve by one day interval.** **A**: field epidemiological investigation, **B**: environmental disinfection.

The 278 cases included 254 students, 18 teachers and 6 school cafeteria food handlers (Table 2). The time distribution showed only 1 peak incidence on November 21^st^ and 22^nd^ (Figure 1). The main symptoms were vomiting (50.0%), abdominal cramps (40.3%), nausea (27.0%), diarrhea (6.8%) and fever (6.8%). The 254 student cases were distributed across 11 grades (Table 3) and 45 classes, including 118 boys and 136 girls (male-to-female ratio = 0.87:1). The attack rates in students from grades 1–6, grades 7–9, and grades 10–12 were 22.0%, 9.3% and 6.0%, respectively, which was significantly different (p < 0.001). Among the other 24 cases, the average age was 35.6 years old (range 29-45 years old).

**Table 2:** **Attack rates of the outbreak across different demographic characteristics**

Characteristics	No. of cases	Total	Attack rate (%)
**Overall**	278	1995	13.9
**Sex**			
Male	125	890	14.0
Female	153	1105	13.8
**Occupation**			
Students in grades 1-6	183	831	22.0
Students in grades 7-9	55	594	9.3
Students in grades 10-12	16	268	6.0
Teachers	18	252	7.1
Cafeteria food handlers	6	50	12.0
**Boarding status (only students)**			
Boarding	223	1373	16.2
Not boarding	31	320	9.7

**Table 3:** **The grade distributions of the student cases in the outbreak**

Grade	Number	Cases	Attack rate (%)
1	182	28	15.38
2	175	30	17.14
3	166	28	16.87
4	158	44	27.85
5	150	53	35.33
6	173	8	4.62
7	171	22	12.87
8	132	15	11.36
9	118	10	8.47
10	94	11	11.70
11	95	5	5.26
12	79	0	0.00
Total	1693	254	15.00

### Laboratory tests

A total of 28 samples were tested positive for *Norovirus* (GII) using real time RT-PCR including 20 rectal swabs and 8 surface specimens. Of the 20 positive rectal swabs, 11 were from student cases and 9 were from asymptomatic cafeteria food handlers who did not report any similar symptoms. Of the 9 asymptomatic food handlers, 7 were engaged in the food distribution and delivery. Among the 8 surface specimens, all of which were from the kitchen, 2 were from kitchen rags, 2 from cabinet handles, 2 from food carts, 1 from the cutlery tray, and 1 from the cabinet’s internal surface. Other samples, including water and food samples, were found to be negative for any bacteria and virus.

### Predominant transmission mode exploring

Due to the positive laboratory results detected on November 22^nd^, the outbreak was hypothesized to be caused by the contaminated cooked food that was distributed and delivered by some asymptomatic food handlers. To verify the hypothesis, attack rates among different groups from November 18^th^-22^nd^ were compared to determine which link on the rectal-oral route was infectious. We collected information on the living conditions, eating conditions and case contact histories of all students. The students who had 3 meals a day were more likely to develop cases than those who only had lunch in the school (Table 4). In addition, the students in grades 1–6 were also more likely to develop cases than the students in grades 7–12.

**Table 4:** **Analysis of relationship between the numbers of meals in school and disease onset among cases from November 18** ^**th**^ **- 22** ^**nd**^ **, 2012**

Category variables	Cases	Total	Attack rate (%)	Chi-square	P-value
Grade (n = 164)					
1-6	133	831	16.0	76.43	<0.001
7-9	27	594	4.5		
10-12	4	268	1.5		
Meals (n = 180)					
Had 3 meals	146	1277	11.4	9.26	0.002
Only had lunch	34	538	6.3		
Boarding or not (n = 164)					
Boarding	142	1373	10.3	3.57	0.06
Not boarding	22	320	6.9		

## Discussion

### Control measures

Due to the infection transmitting around the school, the boarding school was closed from November 22^nd^ to 25^th^. An overall thorough disinfection was conducted in the school cafeteria, classroom building and dormitory building on the afternoon of November 22^nd^. An evaluation of the effect of these continuous disinfection surveillances was performed the following week.

The school sent a notice to all the students’ parents indicating that parents should keep the school informed of whether their children had similar symptoms. In addition, when a new case was reported, he/she should be taken to the hospital for treatment and be required to ask for a 3-day sick leave.

Based on the positive *Norovirus* test result, the asymptomatic food handlers from the school cafeteria were suspected in this outbreak. Those who had 3 meals in the school were more likely to be infected, which may be because they had more chances to be exposed to the infected food. The positive results of both the field investigation and laboratory tests suggested that the hygiene of the school cafeteria was the top concern of this outbreak. Because the cafeteria food handlers were engaged in every cooking procedure, they could easily spread the virus, and we collected rectal swab samples of the remainder of the food handlers in the cafeteria on November 23^rd^ to define the conditions of the asymptomatic infections. Once testing positive, he/she was asked to take a 3-day sick leave. Furthermore, his/her rectal swab needed to have been retested as negative before returning back to work.

After a week of environment surveillance, searching and isolation of infectious cases, including both symptomatic and asymptomatic ones, and thorough disinfection of the school, this outbreak ended, and no new case occurred after December 7^th^.

### Predominant transmission mode

In this outbreak, the shape of the epidemiological curve and laboratory testing outcomes suggested that the most likely transmission mode of *Norovirus* was foodborne. The contaminated agent was *Norovirus* (GII). In addition, based on the exploration of associated factors and positive outcomes in cases, asymptomatic food handlers and in the environments, we identified that the infection may have been transmitted through contaminated cooked food that was distributed by the asymptomatic food handlers. The students who had 3 meals a day in the school were more likely to be infected than those who only had lunch in school. In addition, students in grades 1–6 were also more likely to become cases, because they had meals delivered to their classrooms by food handlers using food carts. In addition, the laboratory test results also proved that there was an infection among food handlers in the school. These findings implied that the asymptomatic food handlers who worked in food distribution and delivery may have caused the disease outbreak. After removing the food handlers, the number of new cases decreased, and the outbreak ended.

### Interpretation of asymptomatic infections

In our study, *Norovirus* was detected from 5 asymptomatic food handlers, and the virus was detected in the rectal swab for 3 weeks from 1 asymptomatic food handler since the first case was detected in 7 days. Our result was consistent with findings in other research. In their studies, Atmar RL et al. and Phillips G et al. found that nearly 30% of the infections were asymptomatic, which may induce a high probability of transmission, leading to an outbreak [13, 14]. Ozawa et al.’s study showed that the prevalence of *Norovirus* infection in food handlers was 19% in different food catering settings in Japan, and the association between *Norovirus* infection in asymptomatic food handlers and disease outbreak was 7% [15]. It was also shown that GII-*Norovirus* infected asymptomatic individuals had mean viral loads similar to symptomatic subjects, indicating a strong probability of transmission in asymptomatic people [16]. However, due to the lack of a culture system or animal model, it was still unclear whether the virus was infectious and how long the infection remained contagious. Although the role of asymptomatic infection in outbreaks was inconclusive, this study addressed an important question regarding when to use public health measures to promptly control *Norovirus*-related outbreaks. Daniels et al. were the first authors to implicate the role of an asymptomatic food handler in an outbreak [17], and they found the same genomic sequence of *Norovirus* in food and a food handler. Ah Yong Jeong et al. also found that the infection of asymptomatic employees as well as symptomatic food handlers may be a potential transmission source in *Norovirus* outbreaks. Given the importance of food handling in preventing *Norovirus* infections, food handlers should be advised to follow good hygiene practices in the kitchen, particularly hand washing.

### Implication of the study

During the outbreak that occurred in this school, positive test outcomes of the 6 food handlers implied a potential association between food handlers and virus transmission. Jun-Hwan Yu et al.’s study found that the excretion of *Norovirus* from asymptomatic food handlers should be an infection source of *Norovirus* outbreaks [18]. They also found that food handlers unrelated to *Norovirus* outbreaks normally can be asymptomatic carriers of *Norovirus*. We did not find a positive RT-PCR outcome in the suspected food, which may be due to the sensitivity of *Norovirus* testing among food samples. We collected the samples from the remainder of the food handlers after the infected food handlers had been identified. Some other studies also suggested this measure to extend the investigation and specimen collection after a food handler was detected as a possible infection source [17, 19]. The food handlers were suggested to not work until 48–72 hours after becoming asymptomatic [4, 14]. However, the shedding period of *Norovirus* has also been found to extend up to 4 weeks [14]. Therefore, the suggestion that leave be approximately 48–72 hours after the disappearance of symptoms may not be sufficient. An evaluation study showed that the transmission was only reduced by 85% through the implementation of these recommended measures. Thus, control measures should be strengthened in order to stop an outbreak quickly. In the present outbreak, the measures were promptly implemented such that each case and asymptomatic cases took a 3-day sick leave. The food handlers could return to work when the laboratory test showed a *Norovirus*-negative result. If the retest was still positive, they would be required to take another 3-day sick leave.

*Norovirus* outbreaks commonly occur in various institutional settings such as schools, daycare facilities, and nursing homes. Health education of hygiene habits is crucial to prevent such outbreaks. A study in elementary schools demonstrated that enhanced hand hygiene and thorough disinfection of surfaces can reduce surface contamination with *Norovirus* [20]. Moreover, all personnel working in the institutional settings for food preparation should receive enhanced training of guidelines, including hand hygiene, masks and gloves wearing, equipment, surfaces and rooms cleaning with an appropriated disinfectant to reduce the possibility of virus transmission in the food chain.

### Strengths and limitations

Our study has added some new insights in the *Norovirus* outbreaks control. It provided a good case to demonstrate the assistance of epidemiological investigation to control an outbreak. Outbreak investigations are important in public health to identify the source, implement control measures, and to prevent future disease. In addition, these investigations frequently generate new knowledge that may amend current control policies [21] and succeed in stopping the outbreak through exclusion of the asymptomatic food handlers and taking prompt control measures prior to further conclusive laboratory evidence. In most areas of China, laboratory testing usually could not be undertaken when an outbreak initially happened, and the epidemiology investigation was the only method to rely on during infectious disease outbreak control. The primary challenge is to stop the outbreak as soon as possible, which is also the basic aim in a local CDC’s field work. Our study provided a practical solution to control the spread of these types of outbreaks when the exact pathogen of the infectious source was unknown. No more new cases were identified as soon as the specific control measures were implemented as suggested by the epidemiology study results. Our experience showed that timely control of the disease outbreak was as important as exploring the disease sources and this approach can be applied in other settings where *Norovirus* outbreaks frequently occur.

The limitations of the study were as follows. First, we just found *Norovirus*-positive results in asymptomatic food handlers and symptomatic cases but not in any of the suspected foods. Without further genomic sequences testing, we also could not draw conclusions based on the RT-PCR outcomes, because we had not found the same sequence among different samples. While challenging, further analysis of genomic sequences is the key to identify the cause in future work. Second, parasites also accounted for some diarrheal cases, especially in children. We did not perform a laboratory diagnosis of parasites such as *Giardia* and *Cryptosporidum* species, which could be potential causative agents and may confound the observed findings.

Third, some biases in our study may have influenced our outcome explanation. For instance, we sent the questionnaire to the students in the school. However, some detailed information on school life and symptoms of elementary school students were provided by their teachers, which may contain observer bias or recall bias.

## Conclusion

This outbreak was a *Norovirus*-associated acute gastroenteritis outbreak. In addition, it was controlled by exclusion of the asymptomatic food handlers and efficient disinfection based on prompt epidemiological investigation. Early identification of the potential cause of this outbreak was necessary to prevent new cases. Further research is needed to resolve the unclear aspects of *Norovirus* transmission; thus, control measures could be adjusted correspondently. Finally, to prevent infection, good hygiene practices such as regular hand washing and efficient disinfection should be promoted.
